# Impact of Cooking Duration on Calcium Oxalate Needle-like Crystals in Plants: A Case Study of Vegetable Taro Flowers in Yunnan

**DOI:** 10.3390/foods13233730

**Published:** 2024-11-21

**Authors:** Haoyu Zi, Rui Chen, Nan Jia, Yuxuan Ma, Chunchang Zhao, Zhe Chen, Jingwei Zhang

**Affiliations:** 1 Yunnan Key Laboratory of Meteorological Disasters and Climate Resources in the Greater Mekong Subregion, Yunnan University, Kunming 650500, China; zihaoyu@stu.ynu.edu.cn (H.Z.); chenrui_7z46@stu.ynu.edu.cn (R.C.); jianan_e0rb@stu.ynu.edu.cn (N.J.); mayuxuan@stu.ynu.edu.cn (Y.M.); 2 Yunnan Key Laboratory of Plant Reproductive Adaptation and Evolutionary Ecology, School of Ecology and Environmental Sciences, Yunnan University, Kunming 650500, China; zhaochunchang@stu.ynu.edu.cn (C.Z.); zhechen2019@ynu.edu.cn (Z.C.)

**Keywords:** taro, taro flowers, vegetable, calcium oxalate, needle crystals, raphides, Yunnan province, heating, economic benefit, Global South

## Abstract

As a popular vegetable in Yunnan Province, China, taro flowers are delicious but contain substances that can cause numbing and mucous membrane damage. Prolonged high-temperature cooking is used by locals to mitigate these effects, though its mechanisms were previously unexplored. This study confirms the presence of needle-like calcium oxalate crystals in taro flowers and shows that prolonged steaming reduces their quantity, size, and sharpness, making them safer to eat. Microscopic observations revealed numerous sharp-tipped (~50 μm) calcium oxalate crystals in fresh taro flowers. After 2 h of steam heating, there were significantly fewer (~80% reduction) and smaller crystals (~70% reduction). Ion chromatography showed no significant change (*p* > 0.05) in calcium oxalate content (remaining ~2.5% of dry weight) after heating. Higher temperatures increase calcium oxalate solubility, causing gradual dissolution and the likely formation of small irregular structures, thus reducing the numbing effect. Prolonged cooking could be applied to other plant-based foods and medicines rich in these crystals. By analyzing statistics related to taro and taro flowers, the estimated potential economic benefits of commercializing taro flowers were USD 2.58–12.92 billion annually, potentially improving food security, creating jobs, and promoting development across regions where taro is largely cultivated in the Global South.

## 1. Introduction

**Figure 1:**
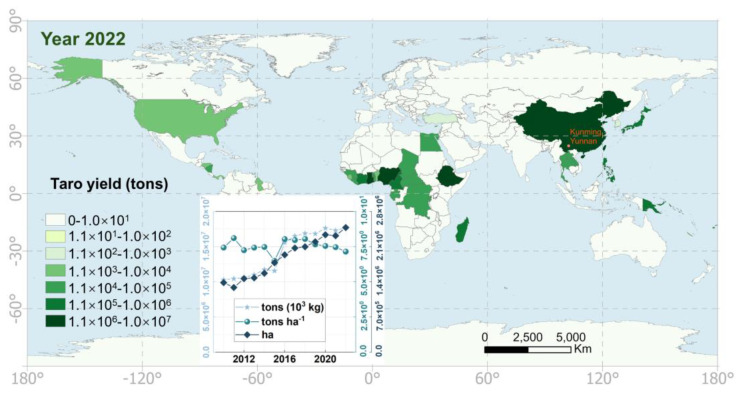
Global taro yield distribution by country (tons) in the year 2022, and the statistics of annual taro yield (ton), yield per hectare (tons ha^−1^), and plant area (hectares, ha) during 2010–2022. The red dot (102.84°E, 24.87°N) denotes the experiment site in this study at Kunming city, Yunnan province, China. Unit conversion explanation: 1 hectare = 100 × 100 m^2^ = 1 × 10^4^ m^2^ = 0.01 km^2^, 1 ton = 1000 kg = 10^3^ kg, 1 ton ha^−1^ = 1 × 10^5^ kg km^−2^. The overlapping in the figure do not affect reading.

Taro (Colocasia esculenta), also known in different regions as cocoyam or papa china, is a perennial plant whose corms are widely consumed [1,2,3,4]. According to statistical data collected from the Food and Agriculture Organization of the United Nations (FAO, https://www.fao.org/ (accessed on 19 November 2024), the global taro cultivation area and production have shown a rapid increasing trend between 2010 and 2022. During this period, the cultivation area expanded from 1.38 × 10^6^ hectares (ha) to 2.48 × 10^6^ ha, while production increased from 1.03 × 10^7^ tons to 1.77 × 10^7^ tons (Figure 1). It is evident that taro cultivation is predominantly distributed across the Global South, including regions such as Africa (e.g., Nigeria, Ghana, and Ethiopia), Asia (e.g., China, Thailand, and Laos), the Pacific Islands (e.g., Papua New Guinea, Solomon Islands, and Fiji), and Central America (e.g., Nicaragua, Dominica, and Honduras) (Figure 1). In these regions, taro corms are utilized in various traditional dishes prepared by boiling, baking, and frying methods [1,2,3,4,5].

**Figure 2:**
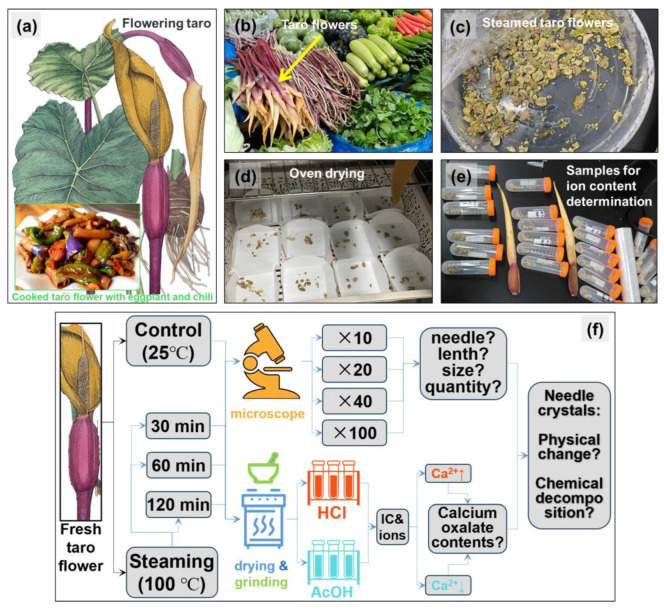
The appearance of flowing taro (*Colocasia esculenta*), including the leaf blade, petiole, corm, and flower, as well as a dish of cooked taro flowers with eggplant and chili in the low left corner (**a**); the taro flowers used in this study, purchased from a nearby market (**b**); appearance of steamed taro flower (**c**); taro flower drying process to obtain the dry weight for each sample by using a hot wind oven (65 °C) (**d**); twenty-four samples collected for subsequent ion determination (**e**); and experimental operation flowchart for this study (**f**).

In most taro cultivation, only the underground corms of taro are harvested and sold, while the aboveground parts are typically discarded as agricultural waste [6], due to their significant needle-like calcium oxalate crystals also known as raphides, which are usually poisonous [7]. Despite this, local residents in Yunnan Province, China, have developed a tradition of consuming taro flowers as a delicacy through long-term practice. Situated in southwestern China and bordering Myanmar, Laos, and Vietnam, Yunnan Province boasts breathtaking scenery. It adjoins the Qinghai-Tibet Plateau to the north, features a tropical rainforest climate in the south, and is home to abundant flora and fauna, including edible floral parts (Figure 1). The taro flower is a cherished vegetable among the people of Yunnan, typically steamed with eggplant and chili pepper, offering a tender, juicy texture and an appealing aroma (Figure 2a) [5].

However, the consumption of taro flowers poses greater risks compared to more common foods. Poisoning from improperly processed taro flowers has occasionally occurred among residents in Yunnan province for decades [8]. Previous studies have reported the formation and distribution of needle-like calcium oxalate crystals in the leaf blade, petiole, and corm peels of taro (the quantity of needle crystals in the inner flesh of the corm is usually low) [7,9,10]. These needle-like calcium oxalate crystals are the main irritating components that can damage oral mucosal tissues upon consumption [6,11]. Additionally, contact with these calcium oxalate needle-like crystals can induce skin irritation [12]. Therefore, the stimulation and poisoning caused by taro flowers are likely due to these needle-like crystals.

Needle-like calcium oxalate crystals in plants serve as a defense mechanism to reduce herbivory [11,13,14,15,16]. These crystals are composed of calcium oxalate monohydrate, i.e., ${CaC}_{2}O_{4}\cdot H_{2}O$ [17,18,19]. High concentrations of calcium oxalate needle-like crystals not only affect the taste, but also cause damage to the digestive system of consumers. Previous studies have shown that calcium oxalate crystals are prevalent in plants from the Araceae family and exist in various forms [14,15,18,19,20,21,22]. However, knowledge about taro flowers, which are traditionally consumed in Yunnan Province, remains considerably limited. As the flower buds of taro, taro flowers possibly contain abundant calcium oxalate needle-like crystals, similar to those found in other parts of taro. Currently, understanding of taro flowers is primarily anecdotal, with a common belief that prolonged high-temperature cooking (such as boiling or steaming at 100 °C) can eliminate the characteristic “numbing sensation”. This perception lacks scientific validation and calls for systematic investigation. To date, there has been no study reporting the presence of calcium oxalate in taro flowers to the best of our knowledge. A search on Web of Science using the keywords “taro flower” and “calcium oxalate” yielded no relevant literature.

From a dietary standpoint, aside from Yunnan in China, no other countries have yet explored the potential of taro flowers as a vegetable. An academic search using Web of Science, along with inquiries on Bing and Google for “taro flower,” revealed an absence of publications or articles discussing the processing of taro flowers as a vegetable outside of Yunnan. However, some regions within Yunnan Province are actively promoting the cultivation of taro flowers. For instance, Jinggu County in Yunnan (100.70° E, 23.49° N) has been highlighted in local government promotional news for its experience in developing the taro flower cultivation industry (https://www.jinggu.gov.cn/info/68506/234551.htm (accessed on 19 November 2024)). In Jinggu County, the flowering period for taro extends from late February to late June. With continuous harvesting, the yield per hectare can reach 15 tons ha^−1^ under careful cultivation, about twice the global mean yield for taro corms (~7.4 tons ha^−1^). The market price ranges between 15–20 CNY Kg^−1^ for taro flowers, corresponding to 2–3 USD Kg^−1^; in contrast, the corms typically sell for ~1 USD Kg^−1^ in Yunnan.

Additionally, the cultivation of taro flowers requires significant labor at every stage. According to local government reports in Jinggu, 20 to 40 local residents were hired daily for tasks such as management, harvesting, and packaging of taro flowers within an area of three hectares in a Jinggu taro farmland. This initiative provides nearby residents with employment opportunities close to home, thereby boosting their incomes and contributing to their prosperity. Furthermore, it plays a crucial role in driving local economic development. For countries in the Global South where taro is widely cultivated (Figure 1), this serves as a valuable reference.

This study explores, for the first time, the characteristics of calcium oxalate in taro flowers during cooking. Both morphological observation and ion concentration determination, focusing on calcium oxalates, were conducted. This research aims to elucidate how heating improves the edibility and flavor of taro flowers, reduces numbness, and lowers poisoning risks. Previous studies have primarily examined calcium oxalate content in fresh taro plants, such as Vietnamese taro leaves [23], and Japanese taro leaf blades and corms [7]. However, there is limited research on changes that these crystals undergo during cooking. The results of this study can serve as a reference for processing other food and medicinal plants rich in calcium oxalate needle-like crystals.

Additionally, taro flowers have been recognized as an excellent edible vegetable by tens of millions of residents in Yunnan. Beyond harvesting the underground corms, the aboveground taro flowers, which were previously discarded, present a promising food source. This development carries significant implications for regions with extensive taro cultivation in the Global South. It has the potential to bolster food supplies, generate employment opportunities, and drive economic growth. Hence, this study provides an opportunity to highlight the potential of taro flowers as a valuable vegetable to food industry professionals globally.

In summary, this study has two aims:

(1) To determine whether taro flowers contain abundant needle-like calcium oxalate crystals similar to other parts of the taro plant, and to examine how these crystals and the total amount of calcium oxalate change with extended cooking time.
(2) To explore the potential for developing and utilizing taro flowers as a vegetable on a global scale, thereby contributing to food security, employment, and development.

## 2. Materials and Methods

### 2.1. Taro Flower Samples and Treatments

Three kilograms of fresh taro flowers were purchased from the nearby farmer’s market in Kunming (102.84° E, 24.87° N), Yunnan Province, southwestern China (Figure 2b). The plant samples were initially rinsed with tap water, followed by a thorough wash with deionized water, and then drained to remove surface moisture. Subsequently, the samples were cut into approximately 1 cm long pieces using scissors.

The prepared taro flower samples were divided into two groups, each placed on three separate trays. Three trays containing fresh samples served as the blank control group and were kept at room temperature of 25 °C in the laboratory. The other three trays were subjected to different steaming durations to analyze the effect of heating time (Figure 2c), covered with plastic wrap, and steamed over high heat (100 °C), with sampling times set for heating durations of 0.5 h (30 min), 1 h (60 min), and 2 h (120 min), respectively. Using three trays for each treatment ensures the execution of three parallel experiments, thereby guaranteeing the reliability of the data.

### 2.2. Observation of Calcium Oxalate Crystals and Effects of HCl and AcOH Treatments

Twelve 10 g samples from each group, including both fresh and those subjected to three different steam-heated durations, were thoroughly ground into a paste using a mortar and pestle. Each treatment included three parallel replicates. The resulting pastes were then uniformly smeared on twelve glass slides. Subsequently, a Leica DM3000 microscope was utilized to examine the presence of needle-like crystals at magnifications of 10×, 20×, 40×, and 100×. The presence, quantity, length, thickness, and other micromorphological characteristics of the needle-like crystals were carefully observed and documented. The length of the crystals was calculated based on the scale bar in the microscope image. First, a ruler was used to measure the length of the calcium oxalate needle crystal in the microscope image. This was then used to measure the length of the scale bar, and the length of the calcium oxalate needle crystal was finally calculated based on the actual length represented by the scale bar.

In addition, the study monitored the variations in the needle-like crystals in fresh samples after adding strong or weak acid solutions. A small drop of either hydrochloric acid or acetic acid solution (1 mol L^−1^) was placed on separate microscope slides with abundant needle-like crystals, to observe the effects of these two acids on the crystals under a microscope. By capturing videos and photographs as the acid solution traversed regions containing crystals, it was possible to determine whether the needle-like crystals were calcium oxalate. This determination was based on its solubility in hydrochloric acid and resistance to dissolution in acetic acid, as referenced in Pueschel and West [24].

### 2.3. Ion Concentration Determination

A total of 24 samples were collected for ion concentration determination (4 durations × 3 parallels × 2 acid treatment = 24), covering fresh samples, and those steamed for 30 min, 60 min, and 120 min. As the experiment progressed, the moisture content of plant samples continuously decreased under steam heating conditions. To precisely determine the ion concentration within the plant material, emphasis was placed on the concentrations of calcium ions and calcium oxalate in the dry weight of the plants. For ion determination, 1.0 g of fresh weight was taken from each sample and then dried to obtain the dry weight. This was achieved using a hot air oven set at 65 °C for approximately 6 h. The samples were weighed every hour, and when the weight remained constant between two consecutive measurements, the samples were considered fully dried (as depicted in Figure 2d,e). This process accounted for variations in water content that might arise from prolonged exposure to high-temperature steam heating in taro flower samples.

Taking advantage of the fact that calcium oxalate is soluble in strong acids but poorly soluble in weak acids [24], equal concentrations (1 mol L^−1^) of dilute hydrochloric acid (Analytical Reagent) and dilute acetic acid (Analytical Reagent) were used for acid treatment. The precise amount of calcium oxalate can be determined by calculating the difference in calcium ion concentration between the two treatments, using the following equation:

(1) $$M_{{CaC}_{2}O_{4}\cdot H_{2}O}=\left(M_{{Ca}_{HCl}^{2+}}-M_{{Ca}_{AcOH}^{2+}}\right)\times \frac{146}{40}$$

where $M_{{Ca}_{HCl}^{2+}}$ and $M_{{Ca}_{AcOH}^{2+}}$are calcium ion concentrations after hydrochloric acid and acetic acid treatments, respectively. The values 146 and 40 represent the molar mass of calcium oxalate monohydrate and calcium ion, respectively. $M_{{CaC}_{2}O_{4}\cdot H_{2}O}$ is the concentration of calcium oxalate (${CaC}_{2}O_{4}\cdot H_{2}O$).

Given the unknown calcium oxalate content in taro flowers, dilute hydrochloric acid was incrementally added to the plant sample in a centrifuge tube, with each addition being 0.5 mL, to ensure the complete dissolution of calcium oxalate present in the sample. The mixture was then examined under a microscope to verify the disappearance of the needle-like crystals. When the volume of added hydrochloric acid reached 2 mL, no needle-like crystals were observed in the sample, indicating that this amount is sufficient to fully dissolve the calcium oxalate. The same volume of 2 mL was also used for acetic acid.

Following the dissolution of calcium oxalate with acid over a 24 h period, the solution was diluted by a factor of 10 by adding 18 mL of water, resulting in a total solution volume of 20 mL for each sample within each centrifuge tube.

Ion chromatography was performed for ion determination by using a Thermo Fisher Scientific Inc. (Waltham, Massachusetts, USA) DIONEX ICS-1100 system. Prior to injection, samples were pre-filtered to remove impurities through a 0.22 μm PTFE syringe filter (ANPEL, SCAA-21) and then diluted 10-fold. The dilution was considered in the final calculation of the calcium oxalate amount, being equivalent to 200 mL of water for each sample.

## 3. Results

### 3.1. Variations in Calcium Oxalate Needle-like Crystals Under Different Cooking Durations

Samples from various stages were ground and applied to slides for microscopic examination, with a zoom range of 10–100× employed to scrutinize the microstructural features of the plant tissues. In fresh plant samples, numerous elongated needle-like crystals approximately 50 μm long, were observed at 10–40× magnification (unbroken crystals after being ground). These needle-like crystals maintained consistent lengths and shapes at both 40× and 100× magnifications, exhibiting extremely sharp ends at 100×. This sharpness likely causes the tingling sensation from tasting fresh taro flowers. These observations are consistent with prior studies on needle-like calcium oxalate crystals in Araceae and other plants, indicating that the crystals present in taro flowers are similarly composed of calcium oxalate [14,18,19,20,21]. Confirmation of the dissolution process was achieved by examining the presence of calcium oxalate crystals before and after exposure to strong or weak acids in subsequent analyses.

**Figure 3:**
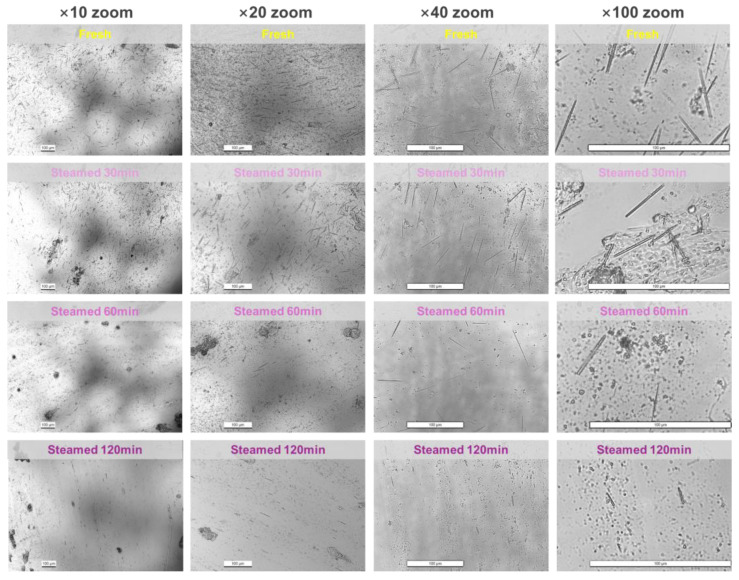
Observed calcium oxalate needle-like crystals under an optical microscope at four magnifications (10×, 20×, 40×, and 100×, from left to right) and at four heating durations (unheated (fresh), steam heated for 30 min, 60 min, and 120 min, from top to bottom).

When samples were subjected to boiling water steam heating at 100 °C, the characteristics of their needle-shaped crystals were tested after heating for 30, 60, and 120 min. After 30 min of steam heating, ~20% fewer needle-like crystals were observed at 10–20× magnification. Higher magnifications (40×) revealed that some crystals gradually became shorter (reduced by ~20%) and thinner, with their sharp ends turning blunt (100×). This reduction in sharpness and size decreased their irritative potential and ability to cause mucosal damage. With continued heating, both the quantity and size of the needle-like crystals significantly reduced. After 120 min of heating, a considerable number of needle-like crystals had disappeared, leaving only a few short, thin needle-like crystals without pointed ends. For example, at 100× magnification, as shown on the right side of Figure 3, the numbers of needle-like crystals in fresh samples and those heated for 30, 60, and 120 min were 13, 7, 5, and 3. The corresponding average lengths were 37.4 ± 9.35 μm, 25.69 ± 7.70 μm, 20.58 ± 11.47 μm, and 9.93 ± 2.96 μm. It is worth noting that some needles physically broke and became shorter after grinding rather than heating.

Hence, prolonged heating effectively reduces the number of needle-like crystals in taro flowers, shortens their length, and blunts their ends, thereby diminishing their irritative properties. Specifically, the length was reduced by ~80%, and the numbers were reduced by ~70% after 120 min of heating.

### 3.2. Changes in Needle-like Crystals Under HCl and AcOH Treatments

Two freshly ground taro flower samples were selected and spread onto microscope slides. Once prominent needle-like crystals were identified under microscopic examination, a small drop of diluted hydrochloric acid (HCl) or acetic acid (AcOH), each at a concentration of 1 mol L^−1^, was applied adjacent to the crystals. Subsequent observations were made to document any changes in the crystals.

**Figure 4:**
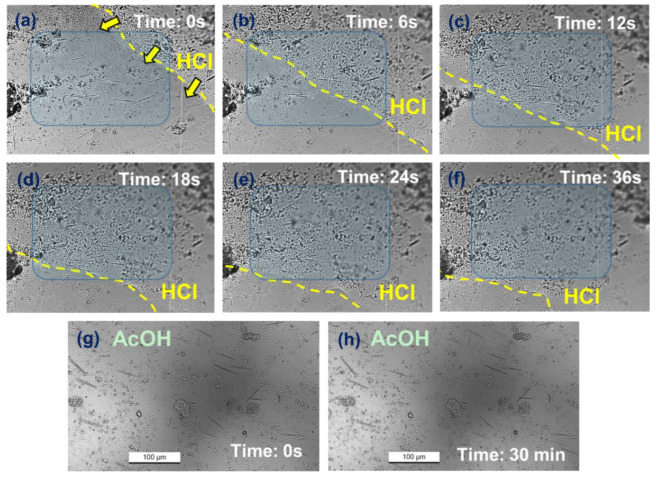
Variations of needle-like calcium oxalate crystals after the addition of HCl (**a**–**f**) and AcOH (**g**,**h**). The yellow dashed line and the arrows indicate the movement of HCl solutions, while the blue background highlights areas concentrated with needle-like crystals. Each panel includes a timestamp displayed in white text.

For the hydrochloric acid-treated samples (Figure 4a–f), it was observed that the acid progressively migrated from the upper right to the lower left. As the diluted acid flowed and permeated the plant sample, the needle-like crystals progressively dissolved within approximately 10 s.

In Figure 4a, the edge of the dilute HCl solution (indicated by yellow dashed lines) had just reached the upper right corner of the area where needle-like crystals were concentrated (light-blue background). At this point, there were about 16 needle-like crystals within the blue frame. After 6 s, the acid traversed roughly half of the blue region, causing the initially contacted crystals to become blurred and start dissolving, although their basic elongated shapes remained visible. Subsequently, as the acid continued to spread, the dissolution of the needle-like crystals progressed. By 24 s, the acid had completely covered the light blue region. Except for a few needle-like crystals in the lower left corner, which were still reacting, most needle-like crystals had nearly vanished, leaving around three discernible ones within the blue frame. By 36 s, almost all needle-like crystals within the blue frame had fully dissolved. These observations indicate that needle-like crystals are readily soluble in dilute hydrochloric acid.

Conversely, for the acetic acid treatment (Figure 4g,h), no change was found in the needle-like crystals after adding the acid. Even after an extended period of 30 min, the number and morphology of the needle-like crystals remained essentially unchanged (Figure 4g,h), with only minor positional shifts attributed to liquid flow.

In summary, the fact that needle-like crystals dissolve quickly in strong acids but not in weak acids further confirms that the primary component of the needle-like crystals is calcium oxalate [24]. This finding is consistent with previous studies on other taro organs such as leaf blades, petioles, and corm peels [7,9,10,23]. The content of calcium oxalate, which is insoluble in water, can also be obtained by measuring the difference in calcium ion content after treating the sample with two different acids, and calculated using Equation (1).

### 3.3. Ion Concentrations and Calcium Oxalate Contents Under Different Cooking Durations

The values of dry weight for the 24 samples are given in Table 1, which includes the grouping of samples, the fresh and dry weights of each group of samples, and the type and amount of acid used in the treatment.

**Figure 5:**
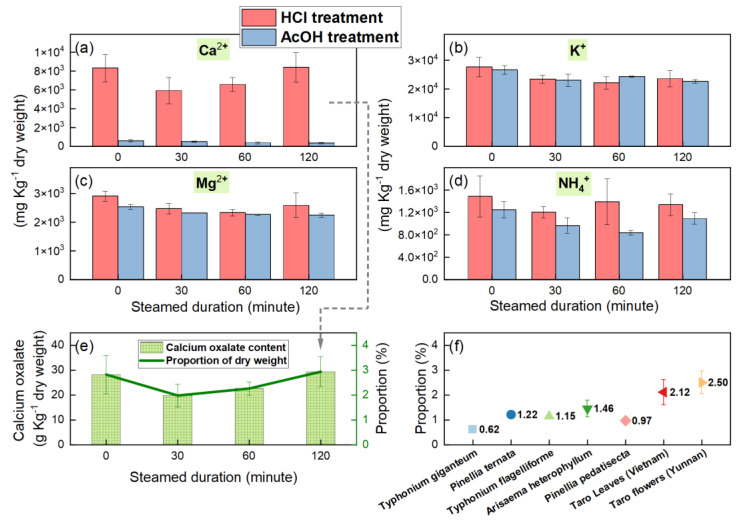
Calcium ion (Ca^2+^), potassium ion (K^+^), magnesium ion (Mg^2+^), and ammonium ion (NH_4_^+^) concentrations for fresh (0 min) and steamed (30, 60, and 120 min) taro flowers with hydrochloric acid (HCl) or acetic acid (AcOH) treatments (**a**–**d**), the calculated calcium oxalate content (in the form of CaC_2_O_4_·H_2_O) in taro flowers, obtained through the difference in calcium ion concentrations via panel (**a**) and Equation (1) (**e**), and a comparison of previously reported calcium oxalate content in Araceae plants with taro flowers reported by this study (**f**).

The ion concentrations were measured by ion chromatography, with the units standardized to mg Kg^−1^ dry weight. The ions analyzed included calcium (Ca^2+^), potassium (K^+^), magnesium (Mg^2+^), and ammonium (NH_4_^+^) cations, under independent treatment conditions using hydrochloric acid and acetic acid (Figure 5a–d). For calcium ions (Figure 5a), it was observed that the calcium content in taro flower samples treated with hydrochloric acid consistently exhibited significantly higher concentrations compared to those treated with acetic acid. The mean values over four timestamps during the experiment were 7313.43 ± 1081.21 mg Kg^−1^ dry weight for hydrochloric acid treatment and 454.13 ± 96.55 mg Kg^−1^ dry weight for acetic acid treatment. Following hydrochloric acid treatment, the calcium concentration increased dramatically by a factor of 15.10 which can be attributable to the dissolution of calcium oxalate. This indicates a low proportion of soluble calcium (6.21%) and a high proportion of insoluble calcium (93.79%) in taro flowers. T-tests confirmed significant differences in calcium ion concentrations between the two acid treatments (*p* < 0.05). However, there were no significant differences in calcium ion concentrations across the four timestamps within each of the hydrochloric acid and acetic acid treatments (*p* > 0.05).

For the other ions (Figure 5b–d), compared to acetic acid treatment, hydrochloric acid treatment resulted in a 30.92% average increase in ammonium concentration (*p* < 0.05). It also slightly increased magnesium concentration by approximately 10%, possibly from the minor decomposition of magnesium carbonate. However, it had a negligible effect on potassium concentration, with a difference of less than 0.2%.

Using the difference in calcium ion concentrations between the two acid treatments and Equation (1), the content of calcium oxalate (in the form of CaC_2_O_4_·H_2_O) was calculated (Figure 5e). The concentrations in the fresh state and after heating for 30, 60, and 120 min were 28.24 ± 7.63, 19.83 ± 4.66, 22.66 ± 2.70, and 29.42 ± 6.01 g Kg^−1^ dry weight, respectively, corresponding to proportions of 2.82 ± 0.76%, 1.98 ± 0.47%, 2.27 ± 0.27%, and 2.94 ± 0.60% (Figure 5e). T-tests revealed no significant differences in calcium oxalate content or its proportion across fresh and various heating durations (*p* > 0.05), suggesting that prolonged steam heating did not lead to the decomposition of calcium oxalate. Considering that calcium oxalate monohydrate decomposes only at temperatures above 200 °C [17,25], it remains in this form in taro flower samples.

In summary, prolonged high-temperature steam heating (100 °C) does not reduce calcium oxalate content (Figure 5e) but significantly decreases the total amount and size of calcium oxalate needle-like crystals (Figure 3). This process transforms their sharp ends into blunt ones, and reduces their sizes and quantities, thereby reducing their irritancy. This finding aligns with the practice of local residents in Yunnan, who cook taro flowers for extended periods to mitigate irritation.

## 4. Discussion

### 4.1. Possible Reasons for the Reduction in Calcium Oxalate Needle Crystals with Cooking

This study observed that with extended heating time, the calcium oxalate needle-like crystals significantly decrease in size and quantity. However, from the perspective of ion concentration changes, the total content of calcium oxalate in taro flower samples did not change significantly (*p* > 0.05). It is hypothesized that increased temperatures enhance the solubility of calcium oxalate crystals, resulting in their dissolution and a consequent decrease in size and quantity. A literature review reveals that the solubility of calcium oxalate increases linearly by about 30% when the temperature is elevated from 25 °C to 90 °C [26], which corroborates the hypothesis.

Despite the enhanced dissolution of calcium oxalate with increased temperature, the increase in solubility is relatively modest (by ~30%). As a result, the dissolution process remains slow even at high temperatures, such as 100 °C during boiling or steaming. This explains why significant amounts of needle-like crystals persist after 30 min of steam heating and only show noticeable reduction and shrinkage after about 1–2 h of prolonged steam heating.

### 4.2. The Potential Recrystallization Process of Dissolved Calcium Oxalate Needle Crystals

The high-temperature steam heating method used in this experiment prevents losses due to dissolution into water, making it representative of the traditional cooking methods commonly employed in Yunnan for preparing taro flowers. The findings of this study indicate that the calcium oxalate content remains predominantly stable throughout conventional cooking methods (Figure 5). The disappearance of calcium oxalate needle crystals, despite their unchanged content, is a fascinating phenomenon. It is hypothesized that rapid cooling after removal from the evaporator causes the saturated calcium oxalate solution to precipitate numerous non-irritating small crystals rather than slowly recrystallizing into needle-like forms.

Generally, when a supersaturated solution undergoes slow cooling, its solubility decreases gradually, causing excess solute to precipitate [27]. This process allows solute molecules to align orderly, forming larger and more regular crystals that are purer due to minimized impurity incorporation [27,28]. Industries such as sugar refining and pharmaceutical manufacturing use slow cooling to produce high-quality large crystals, while laboratories apply this method for controlled studies of crystal morphology and size. In contrast, rapid cooling results in a swift temperature drop, drastically reducing solubility and instantly precipitating the solute. Limited molecular mobility during rapid cooling leads to numerous small, irregular crystals with more impurities [27,28].

For calcium oxalate, rapid cooling and the swift release of supersaturation result in quick nucleation, which probably lead to smaller crystal particles rather than needle-like crystals, in accordance with the basic principles of crystallization from a saturated solution [27,28]. Due to experimental limitations, scanning electron microscopy (SEM) was not performed. While optical microscopy provided length variation characteristics of the needle crystals, it could not accurately measure their width or volume. This limitation will be addressed in future studies.

### 4.3. Comparison with Other Araceae Plants That Have High Calcium Oxalate Content

To further understand the characteristics of calcium oxalate content in taro flowers, a comparison was made with data from relevant literature on typical Araceae plants (Figure 5f). The results show that taro flowers had a relatively high calcium oxalate content, averaging 2.50% of dry weight, slightly higher than the average found in taro leaves in Vietnam, which is 2.12% [23]. Additionally, it exceeds the levels found in some traditional medicinal Araceae plants such as Arisaematis Rhizoma, Typhonium giganteum, Typhonium flagelliforme, Pinellia pedatisecta, and Pinellia ternate, which generally range between 0.6 and 1.5% [29,30]. It should be noted that the samples analyzed in this experiment also included the spathe and stamen parts of taro flowers (Figure 2c). In traditional cooking practices in Yunnan Province, the stamen is generally considered to have a stronger numbing sensation and lower edible value, and there is a preference for consuming the petiole part of the flower, as shown in the bottom left corner of Figure 2a. Therefore, the results of this study might overestimate the overall content of calcium oxalate contents in taro flowers. However, this does not affect the main conclusions of this paper.

Different plants exhibit variations in the morphology of calcium oxalate crystals, such as needle length, sharpness at both ends, and the presence of barbs. These differences can significantly impact mucosal irritation and damage. For instance, even with similar levels of calcium oxalate, the short and thick needles found in Rubiaceae plants cause much less irritation and damage compared to the long, thin needles present in Araceae plants [31]. Therefore, while calcium oxalate content serves as a useful indicator of potential irritancy, microscopic morphological analysis offers a more precise assessment [14,20].

### 4.4. The Potential Benefits of Taro Flowers as a Global Vegetable and Their Role in Poverty Reduction

Based on FAO statistics, the average yield of taro over the past decade has been approximately 7.4 tons ha^−1^ globally. According to data from the United States Department of Agriculture (USDA), the average price of taro is approximately 1.45 USD kg^−1^ (source: https://downloads.usda.library.cornell.edu/usda-esmis/files/k35694332/348509606/d791t862r/cpvl0221.pdf (accessed on 19 November 2024)), which aligns closely with prices in China of ~1 USD kg^−1^.

According to statistics from Jinggu County, Yunnan Province, China, efficient field management can increase the yield of taro flowers to 15 tons ha^−1^, roughly twice the global taro yield per hectare (https://www.jinggu.gov.cn/info/68506/234551.htm (accessed on 19 November 2024)). This increase is attributed to improved water and fertilizer management, as well as harvesting practices. Globally, however, due to a focus on taro corms rather than flowers, the per-unit area yield of taro flowers may not be as high. By assuming the yield of taro flowers ranges from 10% to 50% of taro corm yields, the production of taro flowers in 2022 would correspondingly range from 1.77 × 10^6^ to 8.85 × 10^6^ tons.

Despite the fact that in China the price of taro flowers is 2–3 times higher than that of taro corms, due to the currently lower recognition of taro flowers in other regions, their price is assumed to be half that of taro corms, i.e., 0.73 USD kg^−1^. Therefore, the economic value of global taro flower production in 2022 can be calculated as follows:

$$1.77\times 10^{6}\times 10^{3}\times 0.73\;\;=2.58\times 10^{9\;}USD$$

$$8.85\times 10^{6}\times 10^{3}\times 0.73=12.92\times 10^{9\;}USD$$

Thus, the economic value of global taro flower production in 2022 ranged from approximately USD 2.58 to 12.92 billion.

Furthermore, managing and harvesting taro flowers provides additional employment opportunities, requiring 6–13 workers per hectare in Yunnan, China. Assuming that globally, the number of workers needed per hectare for taro flower management is only 20% of that in China, this translates to 1–3 workers per hectare. In 2022, the global area under taro cultivation was approximately 2.48 × 10^6^ ha. Conservatively estimated, the taro flower industry could potentially create an additional 2.48 to 7.44 million employment opportunities worldwide.

The World Bank’s definition of poverty at 2.15 USD per person day^−1^ (https://www.worldbank.org/en/news/press-release/2022/10/05/global-progress-in-reducing-extreme-poverty-grinds-to-a-halt (accessed on 19 November 2024)) equates to an annual income of 785 USD per person. Thus, the potential economic value of taro cultivation could lift an additional 3.29–16.43 million people with no income out of poverty each year. This has significant implications for enhancing food security, boosting economies, promoting employment, and facilitating development in Global South countries where taro is widely cultivated, especially when considering the rapidly expanding global production and cultivation area of taro (Figure 1).

## 5. Conclusions

The underground taro corm is a widely consumed food globally. Between 2010 and 2022, the global cultivation area of taro expanded from 1.38 × 10^6^ ha to 2.48 × 10^6^ ha, with production increasing from 1.03 × 10^7^ tons to 1.77 × 10^7^ tons. Typically, the aboveground parts of the plant—such as leaves, flowers, and petioles—are deemed inedible and often discarded as agricultural waste. However, in Yunnan Province, China, extensive practice has demonstrated that taro flowers can be transformed into a high-quality vegetable, fetching prices 2–3 times higher than those of the corms. When properly processed, taro flowers exhibit a tender, juicy texture and an appealing aroma, surpassing even eggplant in taste and flavor. Nevertheless, thorough cooking is required to eliminate their numbing sensation in the mouth and reduce potential damage to mucous membranes, likely caused by needle-like calcium oxalate crystals. However, the mechanism of this phenomenon in taro flowers has not been previously studied.

This study evaluates the content and morphology of needle-like calcium oxalate crystals in taro flowers for the first time. The results confirmed that a large quantity of needle-like calcium oxalate crystals is present in fresh taro flowers. Prolonged steaming enhances the solubility of calcium oxalate and significantly reduces the quantity, size, and sharpness of the crystals; however, the overall calcium oxalate content remains largely unchanged. The length of the needle-like crystals decreases from about 50 μm in fresh taro flowers to around 10 μm after two hours of steaming, with a ~70% reduction in their numbers. The proportion of calcium oxalate remains fairly consistent, averaging 2.5% in dry taro flowers, regardless of steaming duration. As steaming continues, the needle-like crystals gradually dissolve and probably reform to many tiny irregular crystals after cooking, reducing irritation and making steamed taro flowers safer to eat. Many edible and medicinal plants, especially in the Araceae family, contain abundant calcium oxalate needle-like crystals, which can be reduced via treatment with a prolonged heating method, as this research suggested.

This study also showcases the potential of taro flowers as a vegetable for use by global food industry professionals for the first time. Preliminary estimates suggest that managing and harvesting taro flowers could generate 2.48 to 7.44 million employment opportunities worldwide and add economic value ranging from USD 2.58 to 12.92 billion annually. The resulting economic benefits could lift 3.29 to 16.43 million individuals out of poverty each year. Thus, the cultivation and commercialization of taro flowers hold significant implications for food security, economic growth, job creation, and development across the Global South, where taro is widely cultivated.

## Figures and Tables

**Table 1:** The names of the schemes in each group, the changes in weight before and after drying treatment, and the description of acid treatment. F: control, S: steamed treatment, 30, 60 and 120: steam heating duration (min).

Case	Fresh Weight (g)	Dried Weight (g)	Acid and Amount (1 mol L^−1^)
F-1-HCl	1.003	0.163	HCl 2 mL
F-2-HCl	1.001	0.176	HCl 2 mL
F-3-HCl	0.996	0.181	HCl 2 mL
F-1-AcOH	0.999	0.146	AcOH 2 mL
F-2-AcOH	1.000	0.178	AcOH 2 mL
F-3-AcOH	1.011	0.156	AcOH 2 mL
S-30-1-HCl	1.010	0.200	HCl 2 mL
S-30-2-HCl	1.011	0.180	HCl 2 mL
S-30-3-HCl	0.992	0.186	HCl 2 mL
S-30-1-AcOH	0.992	0.177	AcOH 2 mL
S-30-2-AcOH	0.998	0.196	AcOH 2 mL
S-30-3-AcOH	1.010	0.188	AcOH 2 mL
S-60-1-HCl	1.008	0.197	HCl 2 mL
S-60-2-HCl	1.013	0.191	HCl 2 mL
S-60-3-HCl	0.996	0.218	HCl 2 mL
S-60-1-AcOH	0.999	0.181	AcOH 2 mL
S-60-2-AcOH	1.008	0.180	AcOH 2 mL
S-60-3-AcOH	0.992	0.171	AcOH 2 mL
S-120-1-HCl	1.009	0.225	HCl 2 mL
S-120-2-HCl	1.009	0.241	HCl 2 mL
S-120-3-HCl	0.999	0.235	HCl 2 mL
S-120-1-AcOH	1.007	0.257	AcOH 2 mL
S-120-2-AcOH	1.007	0.242	AcOH 2 mL
S-120-3-AcOH	0.999	0.212	AcOH 2 mL

## Data Availability

Data will be made available upon request to the authors.
